# Screening tools for early neuropsychological impairment after aneurysmal subarachnoid hemorrhage

**DOI:** 10.1007/s10072-019-04159-w

**Published:** 2019-12-04

**Authors:** Ilari M. Rautalin, Martina Sebök, Menno R. Germans, Miikka Korja, Noemi Dannecker, Olivia Zindel-Geisseler, Peter Brugger, Luca Regli, Martin N. Stienen

**Affiliations:** 1 Department of Neurosurgery, University Hospital Zurich & Clinical Neuroscience Center University of Zurich, Zurich, Switzerland; 2 Department of Neurosurgery, University of Helsinki and Helsinki University Hospital, Helsinki, Finland; 3 Neuropsychology Unit, Department of Neurology, University Hospital Zurich & Clinical Neuroscience Center, University of Zurich, Zurich, Switzerland

**Keywords:** Aneurysmal subarachnoid hemorrhage, Functional outcome, Impairment, Modified Rankin scale, Multi-dimensional outcome assessment, Neuropsychological outcome

## Abstract

**Background:**

Although most aneurysmal subarachnoid hemorrhage (aSAH) patients suffer from neuropsychological disabilities, outcome estimation is commonly based only on functional disability scales such as the modified Rankin Scale (mRS). Moreover, early neuropsychological screening tools are not used routinely.

**Objective:**

To study whether two simple neuropsychological screening tools identify neuropsychological deficits (NPDs), among aSAH patients categorized with favorable outcome (mRS 0—2) at discharge.

**Methods:**

We reviewed 170 consecutive aSAH patients that were registered in a prospective institutional database. We included all patients graded by the mRS at discharge, and who had additionally been evaluated by a neuropsychologist and/or occupational therapist using the Montreal Cognitive Assessment (MoCA) and/or Rapid Evaluation of Cognitive Function (ERFC). The proportion of patients with scores indicative of NPDs in each test were reported, and spearman correlation tests calculated the coefficients between the both neuropsychological test results and the mRS.

**Results:**

Of the 42 patients (24.7%) that were evaluated by at least one neuropsychological test, 34 (81.0%) were rated mRS 0—2 at discharge. Among these 34 patients, NPDs were identified in 14 (53.9%) according to the MoCA and 8 (66.7%) according to the ERFC. The mRS score was not correlated with the performance in the MoCA or ERFC.

**Conclusion:**

The two screening tools implemented here frequently identified NPDs among aSAH patients that were categorized with favorable outcome according to the mRS. Our results suggest that MoCA or ERFC could be used to screen early NPDs in favorable outcome patients, who in turn might benefit from early neuropsychological rehabilitation.

## Introduction

Aneurysmal subarachnoid hemorrhage (aSAH) is a life-threatening disease with short- and mid-term mortality of roughly 40% [1]. In recent years, survival rates have increased due to improvements in risk factor control, early diagnosis, and critical care management, as well as in surgical and endovascular prevention of re-bleeding [2]. While the number of aSAH survivors is slowly rising, the assessment of patients with non-fatal outcomes is becoming increasingly relevant [3].

During the hospital period following aSAH, the outcome of survivors is often evaluated only by functional grading scales such as the modified Ranking Scale (mRS) [4] or Glasgow Outcome Scale (GOS) [5]. Despite their advantages in terms of standardizing the outcome assessment, some criticism has been raised regarding their insensitivity to neuropsychological deficits (NPDs) [6–8]. In fact, NPDs are the most common form of disabilities after aSAH; nearly half of the independent patients suffer from these impairments, causing difficulties in activities of daily living (ADLs) and return to premorbid work [9–11]. It has been shown that early initiation of appropriate rehabilitation after aSAH can decrease the rates of disabilities [12, 13]. Nevertheless, early-phase screening tools that include the neuropsychological and psycho-social aspects of patient outcome are rarely conducted in neurosurgical practice or clinical trials [14].

In a recent study, Haug Nordenmark et al. [15] concluded that the majority of early NPDs after aSAH are missed by the mRS, hence additional assessments should be included in neurosurgical practice. We aimed to determine whether two practical screening tools for NPDs can identify early neuropsychological impairments, even among aSAH patients categorized with favorable outcome that might be discharged instead of receiving inpatient rehabilitation. If a screening tool detected NPDs – even in favorable outcome patients – it could be considered for implementation in a routine discharge assessment. Diagnosing NPDs would enable initiating appropriate therapy, thereby decreasing long-term morbidity of aSAH.

## Material and methods

### Patients

We screened a total of 170 consecutive aSAH patients that were treated at our neurosurgical department, and discharged between January 2015 and April 2018. aSAH diagnosis was confirmed with clinical and radiological examinations (computed tomography angiography (CT-A), digital subtraction angiography (DSA), magnetic resonance angiography (MR-A) and/or spinal tap). In order to be included in the study, patients had to be evaluated by the mRS and at least one additional neuropsychological test before the first hospital discharge after aSAH. Neuropsychological screenings were performed after patients had reached a stable condition (e.g. no signs of hydrocephalus, no delayed cerebral ischemia (DCI), no sodium electrolyte disorder), usually in the last days prior to the hospital discharge.

Information about patients’ age, sex, hypertension (yes/no) and smoking (yes/no) was collected prospectively on admission. Information on complications, particularly hydrocephalus, cerebral vasospasm (CVS), DCI and disability outcome (mRS) were extracted from the institutional patient registry [16] or added by their retrospective chart review as needed. Case-severity of aSAH was defined by the World Federation of Neurosurgical Societies (WFNS) grading scale [17] and the amount of cisternal hemorrhage (Barrow Neurological Institute (BNI) scale [18]). In addition, treatment modality (microsurgical (clipping) vs. endovascular (coiling)) was recorded.

Patients were excluded if they had missing relevant data or documented NPDs before aSAH. In addition, those with fatal, traumatic, non-aneurysmal and perimesencephalic SAH were also excluded.

### Outcome assessments

Based on previous literature [14, 19, 20], the analysis focused on NPDs; the mRS, which is currently used as the “gold standard” for disability after a stroke, was used as a reference variable [21]. The mRS and Montreal Cognitive Assessment (MoCA) are both recommended by the National Institute of Health (NIH) and National Institute of Neurological Disorders and Stroke (NINDS) as valid outcome measures after aSAH (common data elements (CDE) working group on unruptured cerebral aneurysms and SAH [22]). All assessments were made when the patients were first hospitalized after aSAH.

At hospital discharge, all patients were graded according to the mRS by resident and faculty neurosurgeons that had been trained and certified in its use [23]. Since fatal aSAH cases were excluded, the mRS was used to classify patients into one of six categories: 0) no symptoms, 1) no significant disabilities, 2) slight disabilities, 3) moderate disabilities, 4) moderately severe disabilities, or 5) severe disabilities. In line with the definition of the ISAT trial, favorable outcome was defined as mRS categories of 0, 1 and 2 [24].

The MoCA-based evaluations were performed by neuropsychologists trained in its use, following the standard operating procedures for test conduction and scoring [25]. The MoCA is a well-studied, single-page screening tool for NPDs after aSAH that takes around 10—20 min to perform and uses a point-based system to evaluate patients’ abilities of executive functions, naming, attention, recall, abstraction and orientation [14, 19, 26, 27]. One additional point is added for patients with fewer than 12 years of education. From a total of 30 points, a score of <26 has been defined as a score that indicates NPDs [28].

The Rapid Evaluation of Cognitive Function (ERFC = Évaluation Rapide des Fonctions Cognitives [29]) is another screening tool for the evaluation of NPDs in the aSAH population. Although it has been studied less than the other screening tools, its reliability (intra-class correlation coefficient = 0.87, *p* < 0.001) and concurrent validity with the Mini-Mental State Examination (MMSE) test has been demonstrated (0.91, p < 0.001) [30]. The test contains 12 items that assess spatial orientation, attention span, immediate and deferred memory, reasoning and judgment, mental calculation, comprehension, repetition, denomination, execution of a written order, apraxia, verbal fluency, visual decoding and writing. From a total of 50 points, a score of <46 has been defined as a score that indicates NPDs [31]. The ERFC-based evaluations were performed by fully-trained occupational therapists.

### Statistical analyses

Baseline patient characteristics were summarized using mean values with standard deviation in continuous variables, whereas categorical variables were reported using frequencies with proportions as a percentage. The differences between included and excluded patients were evaluated using independent t-tests (continuous variables) and Fisher’s exact tests (categorical variables). Descriptive statistics were used to report the proportion of patients categorized with favorable outcome that showed NPDs according to the MoCA or ERFC. In addition, NPDs were investigated separately in groups with good (mRS = 1) and excellent (mRS = 0) outcome. The relationship between the reference variable (mRS) and each of the NPD screening tools (MoCA and ERFC) was calculated with Spearman’s correlation coefficients. The strength of the correlation was categorized as weak (0.10–0.29), moderate (0.30–0.49) or strong (0.50–1.00) [32]. Linearity was analyzed with a linear regression model (Coefficient and 95% confidence interval (CI)), while the influence of age and sex were adjusted with multiple linear regression. Relationships are graphically presented by scatter plots with fitted regression lines and 95% CIs. Analyses were performed with Stata version 14.2 (Stata Corp, College Station, TX). *p* values <0.05 were considered statistically significant.

## Results

Of the 170 aSAH patients, 42 (24.7%) performed at least one out of the two specific NPD tests during hospitalization, and thus were included in our analysis. The specific proportions for the MoCA and ERFC were 18.2% (*n* = 31) and 9.4% (*n* = 16), respectively. On average, the patients included in the analysis showed milder aSAHs (*p* = 0.002), hospitalization periods that were six days shorter (*p* = 0.011), and more favorable hospital discharge status (*p* = 0.024) compared to aSAH patients who were evaluated only by the mRS (Table 1). NPD screenings were performed on average sixteen (interquartile range (IQR) = 13–21) days after aSAH whereas median difference to mRS assessments was two (IQR = 0–5) days. None of the analyzed patients had excellent functional outcome (mRS = 0) at discharge.

**Table 1:** Characteristics of 170 consecutive aneurysmal subarachnoid hemorrhage patients that were discharged after aneurysmal subarachnoid hemorrhage. The table illustrates (by bolded text) that patients included in this study had lighter World Federation of Neurosurgical Societies admission scores and developed hydrocephalus less frequently. Accordingly, their hospitalization was around six days shorter and their discharge status was more favorable

	**No NPD measurement (excluded)**	**MoCA and/or ERFC measured (included)**	**p value**
Age, mean (SD)	55.5 (12.6)	55.8 (11.3)	0.89
Sex, male (%)	47 (36.7)	12 (28.6)	0.36
Hypertension, n (%)	42 (32.8)	16 (38.1)	0.58
Smoker, n (%)	40 (31.3)	19 (45.2)	0.13
WFNS, n (%) I II III IV V	44 (34.4) 24 (18.8) 6 (4.7) 29 (22.7) 25 (19.5)	25 (59.5) 9 (21.4) 2 (4.8) 6 (14.3) 0 (0)	**0.002**
Treatment, n (%) Microsurgical (clipping) Endovascular (coiling)	66 (51.6) 62 (48.4)	17 (40.5) 25 (59.5)	0.22
Cerebral vasospasm, n (%)	49 (38.3)	20 (47.6)	0.37
Delayed cerebral ischemia, n (%)	27 (21.1)	10 (23.8)	0.83
Hydrocephalus, n (%)	84 (65.6)	17 (40.5)	**0.006**
BNI-scale, n (%) 1) 2) 3) 4) 5)	5 (3.9) 10 (7.8) 42 (32.8) 52 (40.6) 19 (14.8)	0 (0) 3 (7.1) 21 (50.0) 15 (35.7) 3 (7.1)	0.26
Length of hospitalization in days, mean (SD)	29.1 (16.1)	23.6 (10.2)	**0.011**
mRS at discharge, n (%) 0–2 (favorable outcome) 3–5 (unfavorable outcome)	78 (60.9) 50 (39.1)	34 (81.0) 8 (19.0)	**0.024**
	***n*** **= 128 (100%)**	***n*** **= 42 (100%)**	

NPD = neuropsychological deficit

MoCA = Montreal Cognitive assessment

ERFC = The Rapid Evaluation of Cognitive Function

WFNS = World Federation of Neurosurgical Societies

BNI = Barrow Neurological Institute

mRS = modified Rankin Scale

### MoCA and mRS

**Fig. 1:**
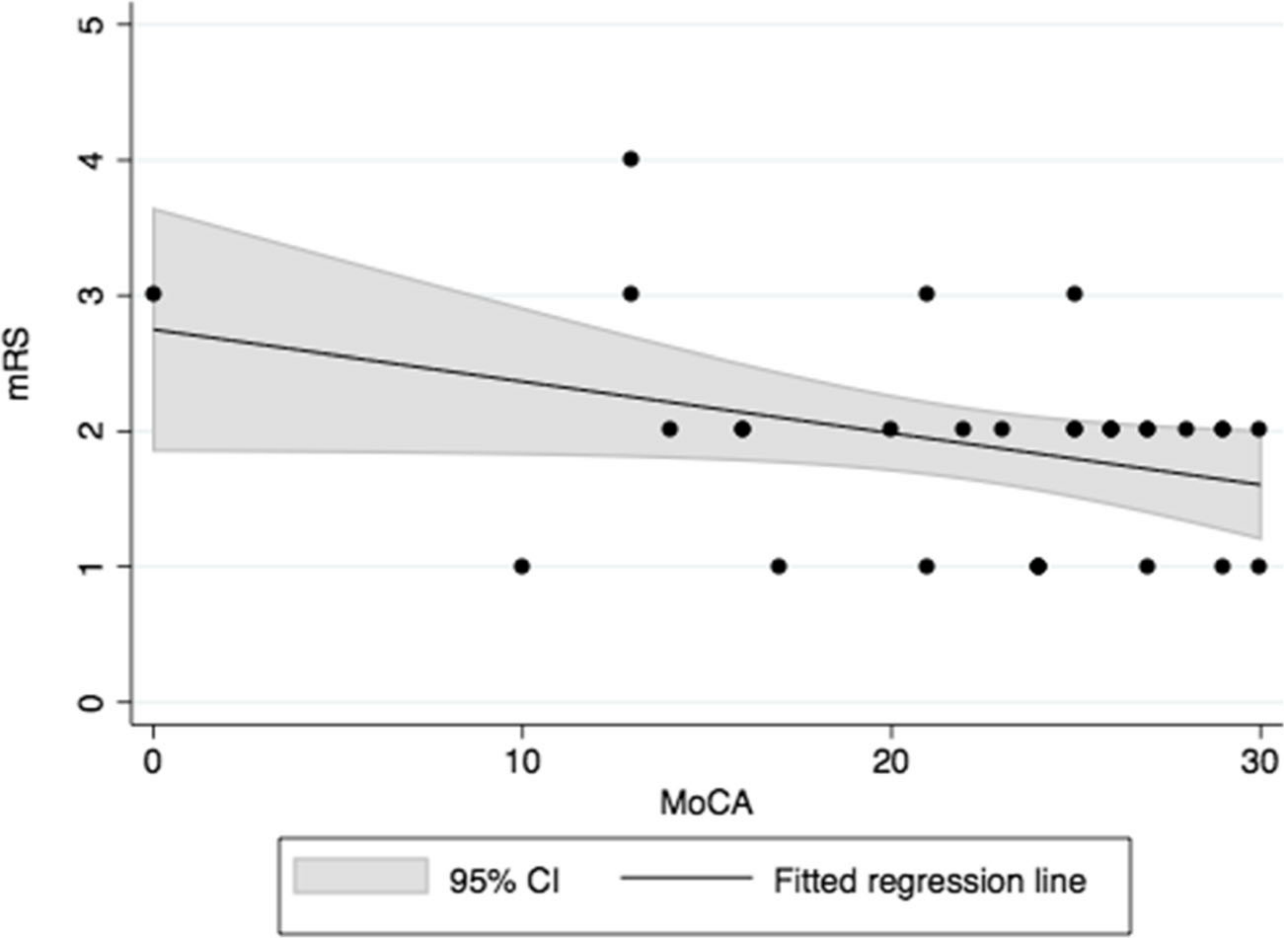
Correlation between the MoCA and mRS

Of the 31 patients with MoCA measurements, nine were categorized with good outcome status (mRS = 1) and 26 with favorable outcome status (mRS = 0–2) at hospital discharge. The prevalence of NPDs was high among these patients: of the nine patients with good outcome and the 26 with favorable outcome, six (66.7%) and 14 (53.8%), respectively, had neuropsychological impairments according to the MoCA. The correlation between the MoCA and mRS was non-significant (Fig. 1; Table 2).

**Table 2:** Correlations between neuropsychological test patterns and the mRS. None of the analyzed patients had excellent outcome (mRS = 0) at discharge

Neuropsychological variable	Number of cases	Correlation coefficients with mRS		Linearity with mRS	Prevalence of NPD in patients with good (mRS = 1) and favorable (mRS = 0—2) outcome, n (%)	
		ρ (95% CI)	p value	p value	mRS 1	mRS 0—2
MoCA	31	−0.24 (−0.62–0.14)	0.208	0.052	6 of 9 (66.7)	14 of 26 (53.8)
ERFC	16	0.050 (−0.46–0.56)	0.849	0.56	7 of 9 (77.8)	8 of 12 (66.7)

### ERFC and mRS

**Fig. 2:**
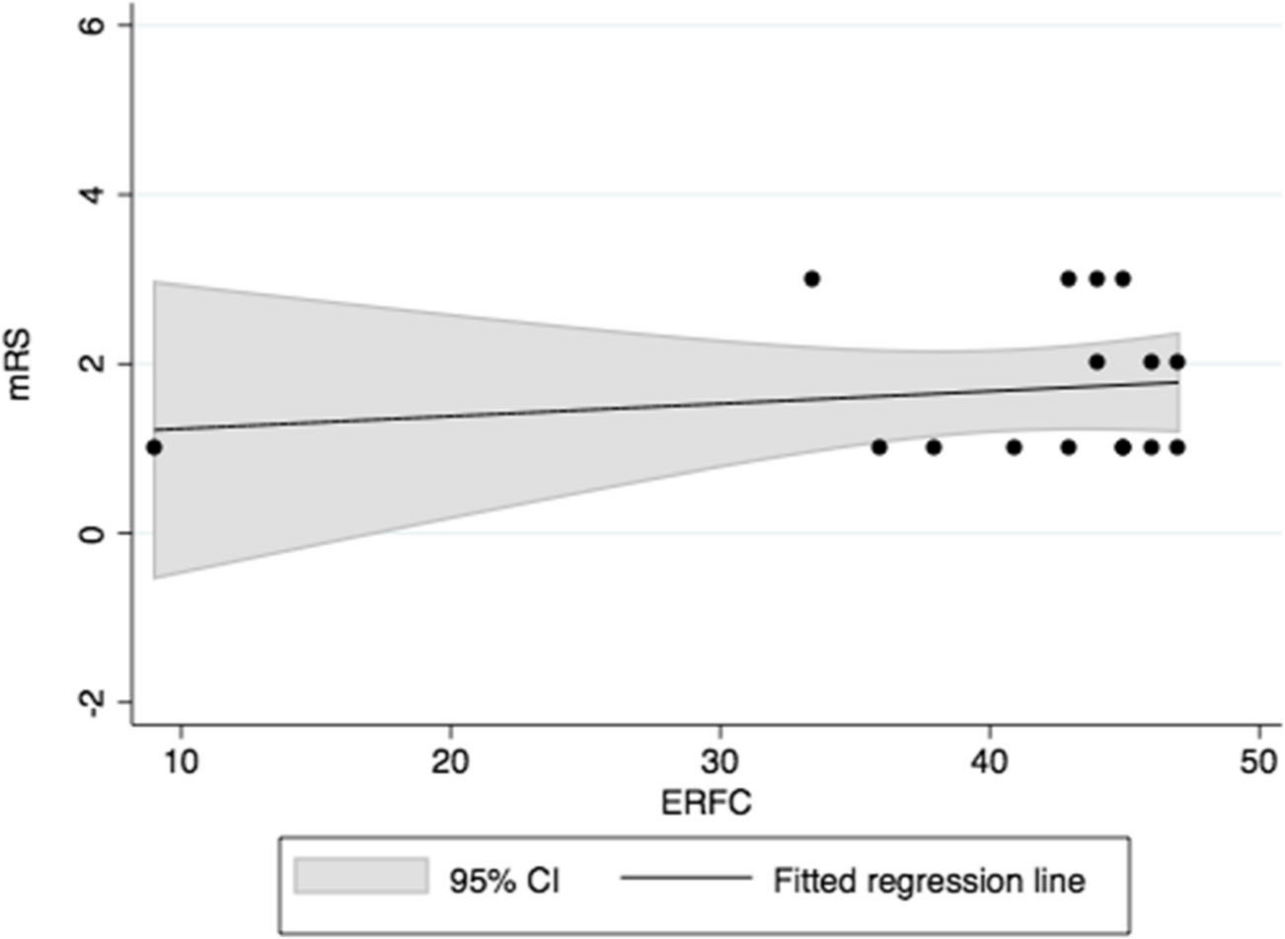
Correlation between the ERFC and mRS

Of the 16 patients with ERFC measurements, nine were categorized with good outcome status (mRS = 1) and 12 with favorable outcome status (mRS = 0–2) at hospital discharge. In line with the results of the MoCA, seven of the nine patients with good outcome (77.8%) and eight of the 12 with favorable outcome (66.7%) had NPDs according to the ERFC. Similar to the MoCA, there was no correlation between the ERFC and mRS, as hypothesized (Fig. 2; Table 2).

## Discussion

The results of this preliminary and retrospective study suggest that the MoCA and ERFC are valuable instruments for the early detection of NPDs in aSAH patients that are in relatively good condition and might be considered for home discharge instead of inpatient rehabilitation. We found NPDs in more than half of the patients that were categorized with favorable outcome according to the mRS (using the described cut-off values for cognitive impairment). NPDs and mRS scores showed no correlation, suggesting that functional and neuropsychological outcomes should be assessed independently. As discharge status and the perceived disabilities at discharge are key indicators for the choice of proper rehabilitation, the simple outcome screening tools examined here would likely help to recognize those aSAH patients who would benefit from a more detailed neuropsychological workup and early neuropsychological rehabilitation.

The favorable outcome status has generally included mRS categories 0–2. However, in order to exclude patients with slight disabilities, a more strict definition has been proposed for this status, which only includes mRS categories of 0 and 1 [33]. Our study shows that the mRS is unable to detect neuropsychological impairments, regardless of the cut-off range. In fact, according to the MoCA and ERFC, the proportions of NPDs were even higher (66.7% and 77.8%, respectively) in aSAH patients with good mRS outcome (mRS = 1). This finding suggests that even when the function of aSAH patients appears similar to the pre-ictal level, simple but validated neuropsychological screening tools can reveal impairments that might otherwise remain unnoticed. Prior works from Haug Nordenmark et al. [15] elucidated a significant mismatch between an – apparently – favorable outcome on the mRS and severely impaired global cognitive function in the acute phase (close to discharge from the hospital). It is possible that with ongoing recovery over time the cognitive scores and disability grading scales align better, however. At three months after the ictus, Wong et al. [27] demonstrated a moderate negative correlation between the mRS and Chinese version of the MoCA (−0.413, *p* < 0.001). One drawback of both our current and the previous study from Haug Nordenmark et al. [15] are the limited sample sizes, which cannot exclude the possibility that the analyses were underpowered to detect a significant correlation. Still, the impressive contrast between relatively mild functional impairment and severely reduced cognitive abilities outlined in both studies leave little room for doubt that the mRS does not qualify to judge upon the need for neuropsychological rehabilitation after hospital discharge.

It has been 25 years since Hütter and Gilsbach highlighted that six months post-aSAH, neuropsychological deficits were frequent even among SAH patients that seemed to fare well. [6] Since then, similar findings have been reported by several other research groups [11, 34, 35]. However, we found only one study that evaluated the incidence of NPDs in patients with favorable functional outcome at the early stages after aSAH. Specifically, Haug Nordenmark et al. [15] reported that 57% of all discharged aSAH patients had a poor cognitive outcome. Similar to our results, nearly half of the cases were still graded with favorable outcome (mRS 0—2). Their conclusion was that in addition to the mRS, another neuropsychological screening tool should be used to identify early NPDs after aSAH. However, the authors did not suggest any specific, short and practical screening tool – which is what we have provided in the present paper.

One of our screening tools, the MoCA, has also been considered by the NIH/NINDS CDE as a first-line neuropsychological screening tool among aSAH patients [22]. The sensitivity for milder NPDs is reportedly high, and the tool has therefore been graded as superior to other commonly used cognitive screening tools, such as MMSE [19, 26]. In fact, in the recent study of 337 aSAH patients, Eagles et al. [35] concluded that although MMSE scores varied in favorable outcome patients (mRS 0—2) 12 weeks after aSAH, more sensitive variables, such as the MoCA, should be used to detect milder NPDs among these patients. Similarly, three studies by Wong et al. have investigated the relationship between the mRS and MoCA [20, 27, 36]. The first study revealed a low association between the MoCA (measured at a subacute phase (2—4 weeks) after aSAH) and unfavorable functional outcome (mRS 3—5) at one year [36]. In the second study, a moderate negative correlation was found between the Chinese version of the MoCA and mRS three months after aSAH [27]. In the third study, only an excellent outcome (mRS = 0) was associated with the MoCA at one year [20]. Similar to our findings, these results emphasize that the MoCA qualifies as a rational tool to screen the neuropsychological function in aSAH patients. Moreover, our ERFC findings are in line with those of the MoCA. However, as the literature behind the ERFC is limited, we believe that more data is needed to confirm its ability to reliably diagnose SAH-associated NPDs.

### Implications for clinical practice and research

After aSAH, NPDs are frequent when routinely screened for, and can encompass executive functions (up to 76% of aSAH patients), memory (up to 61%), language (up to 76%) and sleeping (up to 45%) [10]. These impairments have implications for the affected patients’ ADLs, ability to work and quality of life [9, 10]. Self-reported impairments may be even more frequent: Passier et al. [37] found that 95% of aSAH patients reported cognitive or emotional complaints that caused troubles in ADLs three months after the ictus. Intensive and timely rehabilitation can effectively decrease the amount of dependency and mitigate impairments, even among patient with poor grading at admission [12, 13]. Considering the high prevalence of NPDs as indicated in this study, an appropriate outcome assessment before hospital discharge will likely guide patients to adequate rehabilitation, thereby reducing individual disabilities and positively affecting patients’ return to independent ADLs. Additionally, the reduction of disabilities might mitigate the economic burden of aSAHs, for example by helping otherwise healthy, working-age individuals to return to work [38].

Multidimensional outcome assessment appears similarly important for research: it has been suggested that the lack of appropriate outcome measures is one of the reasons for the failure of many aSAH outcome trials [39]. In addition, it is important to implement a more comprehensive and standardized outcome assessment as early as possible in order to identify patients for effective and early rehabilitation after aSAH. Prospective, multi-center research applying the MoCA as a primary endpoint is currently conducted in Switzerland [25]. In addition, multi-national therapeutic trials enrolling aSAH patients apply neuropsychological screening tools as secondary outcomes [40]. Hence, the implication of a neuropsychological outcome assessment provides the potential for many areas to benefit.

The neuropsychological screening tools presented here may even be routinely used by the neurosurgical staff, namely physicians, physician-assistants or nurses that are trained in the application of such tools. The two screening tools examined in our study appear to have a high sensitivity for detecting NPDs, are relatively fast to conduct, and simple enough to use for screening purposes. Given that neuropsychological resources are limited in the majority of neurosurgical departments globally, an approach that screens aSAH patients with favorable outcome before discharge would seem reasonable. Of course, additional workups by professional neuropsychologists should be organized in cases where the neuropsychological screening is abnormal. Overall, we believe that the population of aSAH patients would benefit from such an approach.

### Strengths and limitations

This study provides new data on the association between the mRS, which is currently the “gold standard” in outcome assessment for stroke and SAH, and more in-depth functional and neuropsychological outcome. It confirms previously reported relationships and adds new data to the existing body of literature. In particular, this study does reconfirm previously described relationships in a modern, contemporary series treated according to modern and potentially less traumatizing endovascular and microsurgical techniques. While the MoCA is a relatively well studied and recommended tool for outcome evaluation after aSAH [22], much less experience has been accumulated for the ERFC in this setting. Similar to earlier studies, the conclusions drawn from our analyses are limited by the small sample size and the retrospective nature of the study. This – and the fact that both the MoCA and ERFC are merely screening tools – is why we desisted from more detailed, domain-specific analysis of NPD, as was done in other studies [15] before for the acute setting. In addition, since all our consecutively admitted 170 aSAH patients were not assessed routinely by NPD screening tools, suspicion of NPDs may have had an effect on the patient selection for additional MoCA or ERFC screening. Nevertheless, the consistency between our results and those from prior studies strengthens our confidence in the findings reported here.

## Conclusion

Neuropsychological impairments were frequently identified among aSAH patients that were categorized with favorable outcome according to the mRS. The current work highlights this previously described phenomenon in a contemporary series of aSAH patients managed according to modern guidelines. Moreover, the data show that by employing a reasonably short and inexpensive neuropsychological screening tool, a substantial proportion of patients is identified who might benefit from early neuropsychological rehabilitation, even though they appear to fare well. Further studies are necessary to evaluate the actual long-term benefit of such an early screening for the patient.
